# Finite element-based prioritization of pelvic floor muscles for rehabilitation to maintain urinary and fecal control in elderly women

**DOI:** 10.3389/fphys.2025.1663545

**Published:** 2025-11-24

**Authors:** Rui Wang, Guangtian Liu, Liwei Jing, Tuanjie Zhao, Xiuqing Qian

**Affiliations:** 1 School of Nursing, Capital Medical University, Beijing, China; 2 College of Nursing and Rehabilitation, North China University of Science and Technology, Tangshan, Hebei, China; 3 Department of Colorectal Surgery, Beijing Erlonglu Hospital, Beijing, China; 4 School of Biomedical Engineering, Capital Medical University, Beijing, China

**Keywords:** elderly women, finite element analysis, muscles, rehabilitation training, urinary and defecation dysfunction

## Abstract

**Objective:**

This study aims to utilize finite element analysis (FEA) to explore the effects of different rehabilitation training methods on the ability of elderly women to maintain urinary and fecal control. It also seeks to determine the muscle prioritization during pelvic rehabilitation training, providing a scientific basis for personalized rehabilitation nursing.

**Methods:**

A 3D pelvic-thigh modeling was constructed based on CT and MRI images from a 70-year-old Chinese elderly female volunteer. Model validity was verified by assessing relative changes in waist circumference, RVA, and ARA against imaging measurements, with geometric deviations controlled within 10%. The material properties of the muscles were altered to simulate the effects of five different physical rehabilitation methods. By comparing changes in the retrovesical angle (RVA) and anorectal angulation (ARA) under different muscle material properties settings, the relationship between rehabilitation training methods and urinary and fecal control was quantified.

**Results:**

The constructed model demonstrated high geometric consistency with pelvic floor anatomy, showing less than 8.28% deviation from imaging-based measurements. As muscle material properties improved, the RVA gradually decreased, and the ARA gradually increased, approaching normal ranges. The results highlight the critical roles of the levator ani, pelvic floor, rectus abdominis, erector spinae, and hip muscles.

**Conclusion:**

The findings from this simulation indicate the potential efficacy of rehabilitation training in supporting urinary and fecal control. The study emphasizes the importance of personalized pelvic floor rehabilitation programs based on gender differences, muscle status, and dysfunction types, offering new perspectives and possibilities for using FEA in elderly populations. Nevertheless, the findings are derived from a single-subject model and computational simulations without direct clinical validation, which may limit generalizability.

**Clinical Trial Registration:**

identifier (ChiCTR2400080749) (20240206).

## 1 Introduction

Our world is becoming increasingly aged, approximately 1.4 billion people aged 60 and above in 2022, and it is expected that this number will double globally by 2050 (WHO, 2022). The growing aging population has exacerbated health challenges related to aging, the overall muscle capacity decreases in elderly individuals, including the pelvic floor, partially leads to the occurrence of urinary incontinence, fecal incontinence, and constipation (Talasz et al., 2018; Ma and Chen, 2020; Levaillant et al., 2023; Salari et al., 2023; Zhang et al., 2023). There are various reasons for elderly people to develop urinary and defecation dysfunction, one of which is that the excretion of feces and urine is controlled and regulated by the neuromuscular system. For elderly individuals who have not completely lost urinary and fecal control, five physical rehabilitation methods, including exercise training, magnetic stimulation, electrical stimulation, vibration stimulation, and biofeedback, have been shown to enhance pelvic floor muscle strength and improve urinary and defecation dysfunction (Elfatah et al., 2023; Alouini et al., 2022; Mazur-Bialy et al., 2020; Brusciano et al., 2020; Höder et al., 2023; Jin et al., 2023; Yang J. et al., 2024).

For muscle rehabilitation training for elderly people with different types of urinary and defecation dysfunction, a “one size fits all” strategy should not be used. Personalization is a extremely important issue (Pinto et al., 2020; Davies et al., 2020; Sarrió et al., 2021; Suhonen et al., 2022). Taking constipation as an example, strategies based on individual risk factors of elderly constipation patients have been proven to reduce the occurrence of constipation (Huang et al., 2015). However, the muscle prioritization for the rehabilitation of elderly individuals with urinary and defecation dysfunction is still not fully understood. It is necessary to explore the quantitative relationship between different rehabilitation training methods and urinary and defecation control ability, in order to provide guidance for personalized rehabilitation training practices.

Finite element analysis (FEA) is an appropriate research methodology for investigating complex relationships between rehabilitation training and pelvic floor function, as it is capable of precisely simulating pelvic floor complex structures and intricate biomechanical behaviors. One of the key advantages of the FEA is that it eliminates the need for human experimentation, thereby ensuring a high level of safety, while also facilitating the execution of numerous experiments. For instance, muscle parameters were varied to observe the corresponding changes in outcome indicators. This approach is not only cost-effective but also highly efficient (Wang et al., 2024a; Chen et al., 2010). As an advanced tool for biomechanical research, FEA has been widely applied in obstetrics and gynecology, highlighting its contributions in simulating pelvic floor dysfunctions (Moseley et al., 2025). Notable studies have simulated female pelvic floor analysis (Xu et al., 2023; Soliman et al., 2023), pelvic organ prolapses in women (Silva et al., 2024; Yang M. et al., 2024; Xue et al., 2023), urethral support functions (Peng et al., 2016), levator ani muscle injuries during vaginal delivery and pelvic floor disorders (Zhou LX. et al., 2020), and the mechanical mechanisms of posterior vaginal prolapse (Qiu, 2017). These previous applications have contributed to the development of pelvic floor simulation models for elderly individuals.

Other studies (Ren et al., 2015; Bhattarai and Staat, 2018; Liu et al., 2022; Gordon et al., 2019) have developed pelvic floor models for female individuals aged 55, 63, and 70 years. However, these existing finite element models are limited to simulating the pelvic floor muscles and tissues neglecting the abdominal, hip, and back muscles, which are critical for rehabilitation training beyond the pelvic floor and play significant roles in urinary and fecal control. Consequently, the current female pelvic floor finite element models offer limited representation of the full spectrum of muscles involved in urination and defecation. Furthermore, these models typically do not address the unique anatomical and physiological characteristics of elderly populations. Few studies have focused on rehabilitation outcomes and the role of muscle prioritization, especially in elderly women with pelvic floor dysfunctions. Therefore, a more comprehensive composite model is needed, one that integrates not only pelvic floor muscles but also abdominal, hip, and back muscles, to provide more accurate rehabilitation guidance tailored for elderly women.

We previously established to establish a finite element model in elderly males, using FEA to explore the quantitative relationship between different rehabilitation training methods and urinary and defecatory control ability (Wang et al., 2024b). The research findings can elucidate the muscle prioritization for rehabilitation training in elderly men suffering from various types of urinary and defecation dysfunction. However, marked physiological and anatomical differences exist between male and female pelvic floors. For example, women have a greater colonic length, a larger rectal cross-sectional area, and lack a prostate but possess female-specific structures such as the uterus, vagina, urethral detrusor muscle and urethrovaginal sphincter. Functionally, females are more prone to deficiencies in the maximum voluntary contraction (MVC) strength and endurance of the external anal sphincter and puborectal muscle, whereas males more commonly exhibit elevated muscle tone and incomplete relaxation (Notenboom-Nas et al., 2023). These differences alter muscle support mechanisms and mechanical load distribution, making it unfeasible to accurately assess female-specific conditions using male model data alone. Therefore, it is necessary to construct a female-specific finite element model to enhance the biological and clinical relevance of pelvic floor simulations. Such a model would allow a direct comparison with male models under the same analytical framework, thereby clarifying sex-based differences in rehabilitation, guiding sex-specific optimization of clinical treatment strategies, and providing more effective personalized rehabilitation plans for elderly patients with urinary and defecatory dysfunction.

This study aims to clarify the relationship between muscle function in elderly women and rehabilitation training, prioritizing the identification of key muscles essential for urinary and defecatory rehabilitation. As early as 2011, Chen et al. (2011) constructed a FEA of the pelvis and levator ani muscles by performing pelvic CT and MRI scans on healthy elderly nulliparous female volunteers. This approach avoided the disadvantage of relying on cadaveric specimen dissection previously used to obtain models. This study aims to collect CT and MRI data from a normal elderly female volunteer and establish a composite finite element model of the pelvic floor. Following the model construction, the study will conduct quantitative validation of the finite element model, to ensure its validity and guarantee the accuracy of the finite element analysis. Similar to the validation method used in the Peng Y’S study (Peng et al., 2016), we compared whether the Valsalva movement process simulated by the patient’s finite element model and the Valsalva movement in the patient’s actual dynamic MRI produced consistent changes (including ARA, RVA, and waist circumference) as a validation method. Muscle material properties, such as the material modulus, are typically related to their physical characteristics. The material modulus describes the ability of muscles to deform under force. Research indicates that the material properties of muscles, including the elastic modulus, may change following rehabilitation training (de Sousa et al., 2023; Peng et al., 2016). Muscle contraction can cause changes in the internal structure of muscles, increasing their resistance to deformation, reflected in an increase in elastic modulus (Morin et al., 2022; Stafford et al., 2017; Aljuraifani et al., 2018; Creze et al., 2018). Under various simulation plans of FEA, the study will proportionally alter muscle material properties to simulate the rehabilitation effects of five physical therapy methods (Peng et al., 2016; Dias et al., 2017; Brandão et al., 2015). For the observed outcome measures, this study selected retrovesical angle (RVA) and anorectal angulation (ARA), which are used to assess the ability of elderly individuals to voluntarily control urination and defecation (Zhou and Lai, 2021; Guo et al., 2015). Among them, RVA is the angle formed between the base of the bladder and the long axis of the urethra in the mid-sagittal plane, and ARA is the angle between the longitudinal axis of the anal canal and the posterior wall of the rectum above the levator ani muscle. Ultimately, the study aims to quantify the relationship between rehabilitation training methods and urinary and fecal control by comparing the changes in outcome indicators under different muscle material properties parameter settings. The results of this study will provide answers to our research question regarding how to implement personalized and targeted rehabilitation training for elderly individuals with, taking into account different genders and types of conditions urinary and defecation dysfunction.

## 2 Methods

### 2.1 Muscles

After a thorough literature review, we summarized the muscles targeted by five rehabilitation training methods for urinary and defecation dysfunction, which include the pelvic floor muscles, core abdominal muscles, and core hip muscles (Table 1).

**TABLE 1:** Muscles corresponding to five rehabilitation training methods for urinary and defecation dysfunction.

Types	Rehabilitation training	Muscles
Urinary incontinence	Pelvic floor muscle training	1. Pelvic floor muscles training: pelvic floor muscles group; Urethral sphincter and levator ani muscle 2. Suspension exercise training: pelvic floor muscles + urethral sphincter and abdominal muscles and hip muscles + back muscles 3. Hip muscle exercise: pelvic floor muscles group and hip muscles
	Electrical stimulation	Pelvic floor muscles group
	Magnetic stimulation	Pelvic floor muscles group
	Biofeedback	Pelvic floor muscles group; levator ani muscle
	Vibrational stimulation	Pelvic floor muscles group
Fecal incontinence	Pelvic floor muscle training	Pelvic floor muscles group; pelvic floor muscles group and external anal sphincter
	Electrical stimulation	Sacral nerve anterior root electrical stimulation: pelvic floor muscles group; pelvic floor muscles group and external anal sphincter
	Magnetic stimulation	Pelvic floor muscle group; pelvic floor muscles group and abdominal muscles + back muscles
	Biofeedback	Pelvic floor muscles group; pelvic floor muscles group and external anal sphincter
	Vibrational stimulation	None
Constipation	Pelvic floor muscle training	Pelvic floor muscles group; levator ani muscle and external anal sphincter
	Electrical stimulation	Pelvic floor muscles group
	Magnetic stimulation	Pelvic floor muscles group; levator ani muscle and external anal sphincter
	Biofeedback	Pelvic floor muscles group; levator ani muscle and external anal sphincter
	Vibrational stimulation	Pelvic floor muscles group

The pelvic floor muscles group includes the bulbospongiosus muscle, ischiocavernosus muscle, superficial transverse perineal muscle, external anal sphincter, deep transverse perineal muscle, urethral sphincter, levator ani, and coccygeal muscle. The levator ani muscle includes the pubococcygeus muscle, iliococcygeus muscle, and puborectalis muscle. In this study, the abdominal muscle specifically refers to the rectus abdominis muscle, while the hip muscles specifically refers to the iliopsoas muscle, quadriceps femoris muscle, gluteus maximus muscle, hamstring muscle, gluteus medius muscle, and adductor longus muscle. In this study, the back muscle specifically refers to the erector spinae muscle.

### 2.2 Participant

**FIGURE 1:**
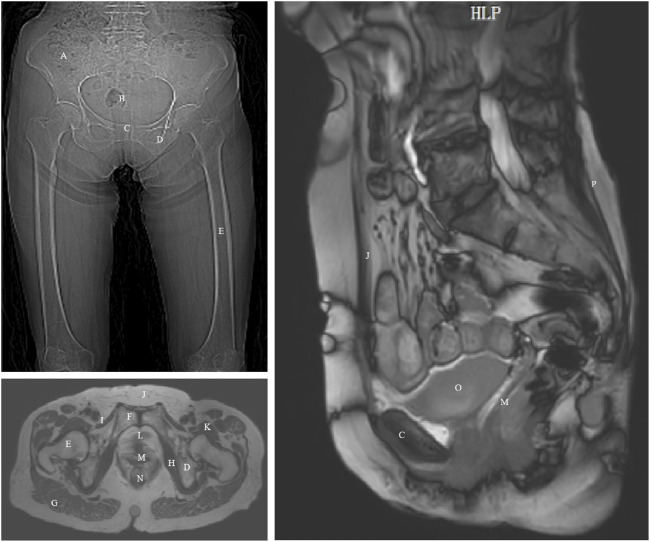
CT and MRI images of an elderly woman with annotated anatomical landmarks. **(A)** ilium; **(B)** sacrum and coccyx; **(C)** pubis; **(D)** ischium; **(E)** femur; **(F)** pubic symphysis; **(G)** gluteus maximus; **(H)** obturator internus; **(I)** pectineus; **(J)** rectus abdominis; **(K)** iliopsoas; **(L)** urethra; **(M)** vagina; **(N)** rectum; **(O)** bladder; **(P)** erector spinae.

Relevant ethical approval was obtained from Capital Medical University (ethics approval numbers: Lin Yan Shen [2023] 079 and Z2024SY007) and the study has been registered with the Chinese Clinical Trial Registry Center (ChiCTR2400080749). This study selected an elderly woman as a typical representative of healthy elderly pelvic anatomy. The participant was a 70-year-old Chinese female (height: 1.56 m, weight: 45 kg, BMI: 18.49 kg/m^2^) with good physical health, normal cognitive, communication, and daily living abilities, normal pelvic floor function, and no history of bowel dysfunction or related surgeries. The volunteer signed an informed consent form before undergoing CT, static MRI, and dynamic MRI scans, as shown in Figure 1. Details regarding the inclusion and exclusion criteria and specific data collection methods are provided in the Supplementary Material of our previously published protocol (Wang et al., 2024a).

The image acquisition was performed after emptying the bladder and rectum, and the participant was able to perform both resting and Valsalva maneuvers under the guidance of professional technicians. Based on the medical imaging data from the elderly female volunteer, we established a 3D modeling of the pelvis and thigh muscles. The method for establishing the finite element model is similar to that described in our previous study (Wang et al., 2024b). The segmentation process was conducted under the guidance of experienced pelvic floor imaging specialists. The researchers first used Mimics 20.0, a medical image processing software, to reconstruct an initial 3D model based on CT and MRI data. This preliminary model was then refined using the smoothing function within Mimics to reduce point and edge angularity and ensure smoother anatomical surfaces. After these adjustments, the final optimized 3D model was exported and imported into Ansys 2020 R1 for subsequent finite element numerical simulations.

### 2.3 Material properties

When setting material properties for the model, we reviewed existing material data from previous studies (Peng et al., 2016; Dias et al., 2017; Brandão et al., 2015; Chen et al., 2015; Li, 2022; Venugopala Rao et al., 2010; Xu et al., 2021; Chen, 2014; Samavati et al., 2015; Boubaker et al., 2009; Sichting et al., 2014; Zong, 2022; He, 2016; Ricci et al., 2018; Guo et al., 2019; Zhang et al., 2009; Ma, 2019; Rao et al., 2010; Silva et al., 2016; Xuan et al., 2021; Lee et al., 2005; Wu, 2019; Krcmar et al., 2015; Liu, 2017; Sun, 2015; Li et al., 2017) and summarized the recognized mechanical parameters reported in the literature (Table 2). Studies have shown differences in the mechanical properties of pelvic soft tissues between elderly and younger populations, with potential variations in material properties (Chantereau et al., 2014). Based on these findings, we made gradual adjustments to the material properties of the pelvic floor muscles to reflect the expected biomechanical changes with aging. These adjustments were made incrementally until the differences between the finite element model and dynamic MRI measurements of ARA, RVA, and waist circumference were within 10%. This process ensured that the model accurately simulated the physiological changes that occur with aging.

**TABLE 2:** Material properties of various parts of the model.

No.	Anatomical element	Material constants		Structures	Constitutive models	
		In the literatures	After adjustment			
1	Bladder	C_10_ = 0.071 C_20_ = 0.202 C_30_ = 0.048 (Chen et al., 2015; Li, 2022)	C_10_ = 0.061 C_20_ = 0.202 C_30_ = 0.048	hyperelastic structures (Chen et al., 2015; Venugopala Rao et al., 2010)	Yeoh (Chen et al., 2015; Li, 2022)	
2	Urethra		C_10_ = 0.071 C_20_ = 0.282 C_30_ = 0.048			
3	Rectum	C_10_ = 0.088 C_20_ = 3.092 C_30_ = 2.871 (Li, 2022)	C_10_ = 0.082 C_20_ = 3.092 C_30_ = 2.871			
4	Uterus	young’s module 2 MPa (Samavati et al., 2015; Boubaker et al., 2009) Poisson’s ratio 0.45 (Samavati et al., 2015; Boubaker et al., 2009)	young’s module 2.5 MPa Poisson’s ratio 0.45	linear elastic structures (Dias et al., 2017)	Hooke (Dias et al., 2017)	
5	Vagina	young’s module 2 MPa (Samavati et al., 2015; Boubaker et al., 2009) Poisson’s ratio 0.49 (Dias et al., 2017)	young’s module 2.8 MPa Poisson’s ratio 0.49			
6	Hipbone	young’s module 15244 MPa (Sichting et al., 2014) Poisson’s ratio 0.3 (Dias et al., 2017; Sichting et al., 2014; Zong, 2022; He, 2016; Ricci et al., 2018; Guo et al., 2019)	No change	rigid body (Brandão et al., 2015; Xu et al., 2021)		
7	Sacrum	young’s module 16262 MPa (Sichting et al., 2014) Poisson’s ratio 0.3 (Dias et al., 2017; Sichting et al., 2014; Zong, 2022; He, 2016; Ricci et al., 2018; Guo et al., 2019)				
8	Coccyx	young’s module 11000 MPa (Sichting et al., 2014) Poisson’s ratio 0.3 (Dias et al., 2017; Sichting et al., 2014; Zong, 2022; He, 2016; Ricci et al., 2018; Guo et al., 2019)				
9	Femur	young’s module 13500 MPa (Guo et al., 2019) Poisson’s ratio 0.3 (Dias et al., 2017; Sichting et al., 2014; Zong, 2022; He, 2016; Ricci et al., 2018; Guo et al., 2019)				
10	Fat	young’s module 0.05 MPa (Dias et al., 2017) Poisson’s ratio 0.49 (Dias et al., 2017; Zhang et al., 2009; Ma, 2019)		linear elastic structures (Peng et al., 2016; Dias et al., 2017; Rao et al., 2010)	Hooke (Peng et al., 2016; Dias et al., 2017; Rao et al., 2010)	
11	Bulbospongiosus muscle	young’s module 2.4 MPa (Peng et al., 2016; Dias et al., 2017; Zhang et al., 2009; Rao et al., 2010) Poisson’s ratio 0.49 (Dias et al., 2017; Rao et al., 2010)	young’s module 3.4 MPa Poisson’s ratio 0.49			
12	Ischiocavernous muscle					
13	Superficial transverse perineal muscle, deep transverse perineal muscle					
14	External anal sphincter	C_10_ = 11.8 KPa C_20_ = 5.53e-3 KPa (Silva et al., 2016)	C_10_ = 12.8 KPa C_20_ = 5.53e-3 KPa	hyperelastic structures (Silva et al., 2016; Xuan et al., 2021)	Mooney-Rivlin (Silva et al., 2016)	
15	Urethral sphincter					
16	Urethral detrusor muscle	young’s module 2.4 MPa (Dias et al., 2017; Zhang et al., 2009) Poisson’s ratio 0.49 (Dias et al., 2017)	young’s module 3.4 MPa Poisson’s ratio 0.49	linear elastic structures (Samavati et al., 2015)	Hooke (Samavati et al., 2015)	
17	Urethrovaginal sphincter					
18	Coccygeal muscle	young’s module 2.4 MPa (Dias et al., 2017; Zhang et al., 2009) Poisson’s ratio 0.49 (Dias et al., 2017; Xu et al., 2021)	No change			
19	Levator ani muscle, including pubococcygeus muscle, iliococcygeus muscle, puborectalis muscle	C_10_= 2.5 KPa C_20_= 0.625 KPa (Lee et al., 2005; Wu, 2019)		hyperelastic structures (Krcmar et al., 2015)	Mooney-Rivlin (Wu, 2019)	
20	Obturator internus muscle	young’s module 0.95 MPa (Peng et al., 2016; Dias et al., 2017; Liu, 2017) Poisson’s ratio 0.45 (Chen, 2014)		viscoelastic materials (Li et al., 2017)	Hooke	
21	Rectus abdominis muscle	young’s module 13.3 MPa (Liu, 2017) Poisson’s ratio 0.45 (Chen, 2014)				
22	Lliac muscle	young’s module 19 MPa (Li et al., 2017) Poisson’s ratio 0.45 (Chen, 2014)				
23	Psoas major muscle	young’s module 13.3 MPa (Sun, 2015) Poisson’s ratio 0.45 (Chen, 2014)				
24	Quadriceps femoris muscle					
25	Tensor fascia lata muscle					
26	Gluteus maximus muscle	young’s module 19 MPa (Li et al., 2017) Poisson’s ratio 0.45 (Chen, 2014)				
27	Gluteus medius muscle					
28	Piriformis muscle					
29	Hamstring muscles	young’s module 13.3 MPa (Sun, 2015) Poisson’s ratio 0.45 (Chen, 2014)				
30	Pectineus muscle					
31	Adductor longus muscle					
32	Latissimus dorsi muscle					
33	Erector spinae muscle					

Due to the complexity and irregularity of structures in the human body, an unstructured meshing approach was adopted for volumetric mesh generation, resulting in a model with a mixed mesh of tetrahedral and hexahedral elements. This hybrid meshing approach leverages the advantages of both element types. Tetrahedral meshes offer greater flexibility in handling complex geometries, while hexahedral meshes generally provide higher computational efficiency and accuracy during numerical simulations. This combination ensures model accuracy while enhancing computational efficiency.

### 2.4 Constraint boundaries

The coordinates for the 3D finite element model were set as follows: the x-axis was perpendicular to the sagittal plane of the body, with the positive direction pointing to the left; the y-axis was perpendicular to the coronal plane, with the positive direction pointing backward; the z-axis was the longitudinal axis of the body, with the positive direction pointing toward the head. To simulate the *in vivo* state of muscles related to urinary and fecal control as accurately as possible, the bones were set as rigid bodies, and all nodes were fully constrained to prevent translation and rotation along the x, y, and z-axes. The tops of the rectus abdominis, psoas major, and erector spinae muscles were fixed, and bindings were established between the bones and muscles, between the bladder and uterus, as well as between the uterus and colon.

### 2.5 Mesh sensitivity analysis

Mesh generation is a key step (Pisarciuc et al., 2023). To verify the sensitivity of the finite element model results to the mesh discretization, a mesh independence study was conducted (Henninger et al., 2010). Under the condition of consistent geometry, material properties, boundary conditions, and load settings, two mesh models with different densities were created: the original mesh density (100%) and a refined mesh density (130%) (Oefner et al., 2021). The same working condition (Condition No.1) was applied to solve both mesh models, and the total deformation cloud maps were output. The relative change rate of the maximum total deformation value was used to evaluate mesh convergence. A relative change rate of 1∼5% was considered to indicate that the model had met the mesh independence verification (Oefner et al., 2021).

### 2.6 Model validity verification

It is essential to verify the effectiveness of the finite element model during the modeling process. In this study, when acquiring dynamic MRI data, the patient was instructed to perform the Valsalva maneuver. The Valsalva maneuver was performed by participants under breath-holding conditions to simulate the increase intra-abdominal pressure for forceful evacuation of bladder and rectal contents, with the sustained effort maintained for 10 s. The results of this maneuver were used for model validation through a geometric verification method. The specific method for geometric validation involved comparing the three anatomical landmarks related to the pelvic floor Valsalva maneuver in the elderly woman during dynamic MRI scanning with those simulated in the 3D finite element model. The relative changes in these three anatomical landmarks between the imaging and simulation were assessed to determine whether they fell within an acceptable range. The three anatomical landmarks used include the abdominal waistline, RVA, and ARA (sagittal plane). For the Valsalva simulation, the loading site was the anterior abdominal wall. Under resting conditions, the material modulus (E) was 0.019 MPa, while the load due to abdominal pressure was 0.5 KPa. Under moderate tension, E was 0.241 MPa and the abdominal pressure was 4.5 KPa. Under high tension, E was 0.947 MPa and the abdominal pressure was 5.0 KPa (Wu, 2019). The applied direction of force was from the top of the anterior abdominal wall toward the coccyx (Qiu, 2017).

### 2.7 Applied loads and simulation plans analysis

The 3D finite element model was used to simulate and analyze the impact of different physical rehabilitation methods on muscle function. Owing to the inability of FEA to directly simulate rehabilitation training, the rehabilitation effects were assessed by adjusting the material properties of the muscles. The elastic modulus, which is the ratio of deformation to applied force, is an important parameter for evaluating muscle stiffness. Studies have shown that rehabilitation exercises, such as muscle contraction training and resistance exercises, can indirectly influence this modulus, leading to a reduction in passive stiffness and an increase in muscle softness and flexibility (de Sousa et al., 2023).

Under different simulation plans, this study proportionally adjusted the material properties of muscles to theoretically simulate changes in muscle function after different rehabilitation training methods (Peng et al., 2016; Dias et al., 2017; Brandão et al., 2015). Specifically, the material constants of the muscles involved in the rehabilitation training were adjusted to 2 times, 1.75 times, 1.5 times, 1.25 times, 1 time, 0.75 times, 0.5 times, 0.25 times, and 0.05 times, simulating muscle capacity changes of +100%, +75%, +50%, +25%, normal, −25%, −50%, −75%, and −95%, respectively. The elastic modulus of pelvic floor and related muscles was varied from 0.05 times to 2.0 times of the baseline values to represent a wide spectrum of physiological conditions ranging from severe impairment to strengthening. This approach has been previously adopted in computational modeling studies. For example, Dias et al. (2017) applied multiplicative factors from 0.05 times to 2.0 times to muscle material properties in their finite element analysis of pelvic floor dynamics during high-impact activities, thereby providing a precedent for this parameter variation strategy.

The relationship between rehabilitation training and the ability to control urinary and fecal functions was primarily quantified by comparing the differences in RVA and ARA using different material properties. This helped in identifying which muscles showed the greatest improvement in urinary and defecation control ability after rehabilitation training (Table 3).

**TABLE 3:** Simulation plans table.

Conditions no.	Simulated rehabilitation trainings	Loading site	Abdominal pressure	Outcome indicators
1	Anal sphincter training for fecal incontinence	External anal sphincter	0.6 KPa (Wang et al., 2024b; Song et al., 2012; Korkmaz and Rogg, 2007)	ARA
2	Biofeedback for urinary incontinence Exercise training for fecal incontinence-levator ani activities Exercise training for constipation-levator ani exercise	Levator ani muscle	0.6 KPa (Wang et al., 2024b; Song et al., 2012; Korkmaz and Rogg, 2007)	RVA, ARA
3	Exercise training for urinary incontinence	Levator ani muscle and urethral sphincter	0.6 KPa (Wang et al., 2024b; Song et al., 2012; Korkmaz and Rogg, 2007)	RVA
4	Magnetic stimulation of constipation Biofeedback of constipation Exercise training for constipation	Levator ani muscle and external anal sphincter	0.6 KPa (Wang et al., 2024b; Song et al., 2012; Korkmaz and Rogg, 2007)	ARA
5	Exercise training for urinary incontinence Electrical stimulation for urinary incontinence Magnetic stimulation for urinary incontinence Biofeedback for urinary incontinence Vibrational stimulation for urinary incontinence Electrical stimulation for fecal incontinence-sacral nerve anterior root electrical stimulation Biofeedback for fecal incontinence combined with pelvic floor muscle training Magnetic stimulation for fecal incontinence Exercise training for constipation-pelvic floor muscle training Tibial nerve electrical stimulation, sacral nerve electrical stimulation, and transcutaneous acupoint electrical stimulation for constipation Magnetic stimulation for constipation Biofeedback for constipation Vibrational stimulation for constipation	Pelvic floor muscles	0.6 KPa (Wang et al., 2024b; Song et al., 2012; Korkmaz and Rogg, 2007)	RVA, ARA
6	Magnetic stimulation for fecal incontinence-functional magnetic stimulation	Pelvic floor muscles and rectus abdominis muscle and erector spinae muscle	0.6 KPa (Wang et al., 2024b; Song et al., 2012; Korkmaz and Rogg, 2007)	ARA
7	Exercise training for urinary incontinence-hip muscle exercises	Pelvic floor muscles and hip muscles	0.6 KPa (Wang et al., 2024b; Song et al., 2012; Korkmaz and Rogg, 2007)	RVA
8	Exercise training for urinary incontinence-suspension exercise training	Pelvic floor muscles and rectus abdominis muscle and hip muscles and erector spinae muscle	0.6 KPa (Wang et al., 2024b; Song et al., 2012; Korkmaz and Rogg, 2007)	RVA

## 3 Results

### 3.1 3D finite element model establishment

**FIGURE 2:**
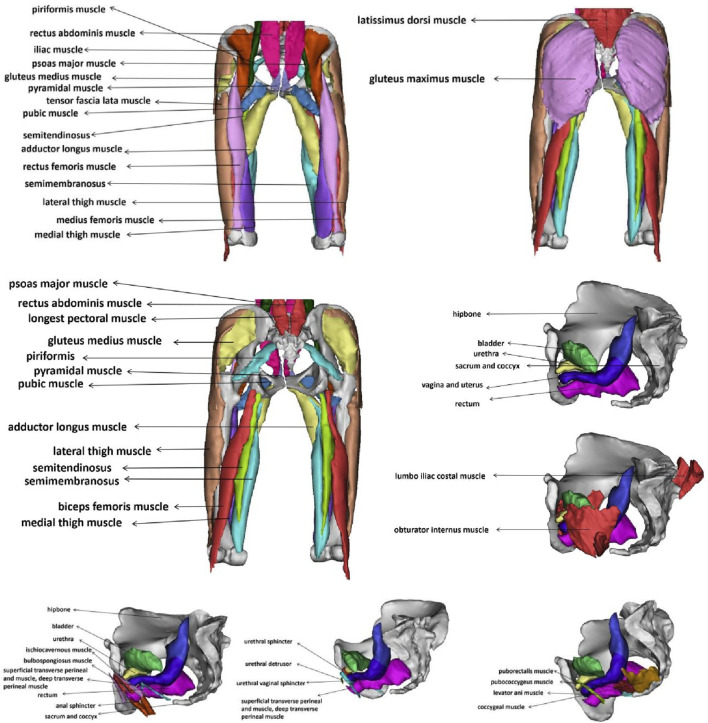
Annotation diagram of different muscle positions of the 3D pelvic-thigh model.

This study successfully constructed a finite element model of the pelvic floor muscles and the hip, abdominal, and back muscle groups related to urinary and fecal functions in elderly women. The final 3D pelvic-thigh model of elderly women includes 43 structures. Figure 2 is an annotation diagram of different muscle positions of the modeling.

### 3.2 Mesh independence and deformation analysis

The total deformation distribution maps under two different mesh densities show that the deformation patterns of the two models are consistent. The maximum total deformation for the original mesh (100%) model was 0.6459 mm, while the maximum total deformation for the refined mesh (130%) model was 0.6269 mm (Supplementary File 1). The relative change between the two was 3.03%, which is less than 5%, indicating that the mesh refinement has a minimal effect on the overall deformation results. The results are not sensitive to mesh density, and the model demonstrates good mesh independence. Therefore, for subsequent calculations, the original mesh model (100%) before refinement will be used as the standard mesh to balance computational accuracy and efficiency.

**FIGURE 3:**
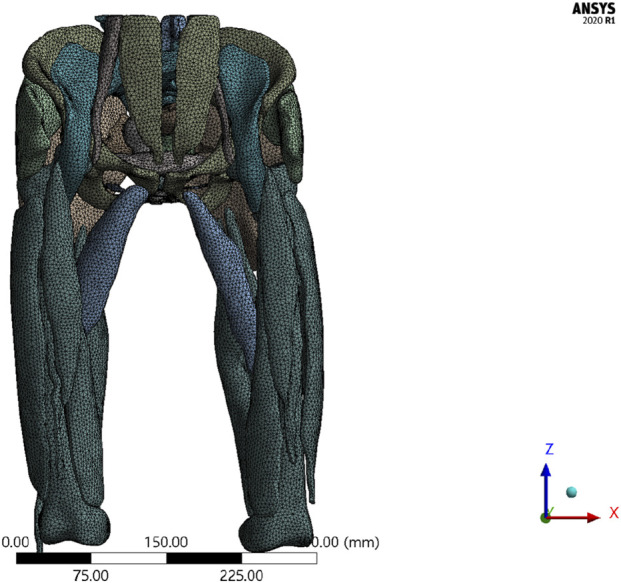
Mesh division of the 3D finite element model.

Figure 3 illustrates the mesh division of the 3D finite element model, comprising 69,635 nodes and 148,161 elements. A hybrid meshing strategy was applied to the composite model using both tetrahedral and hexahedral elements, which optimizes simulation efficiency without compromising anatomical fidelity. This mesh layout ensured accurate force transmission across the integrated pelvic-thigh model and facilitated subsequent load simulations under different rehabilitation scenarios.

### 3.3 Verification of 3D finite element model

**FIGURE 4:**
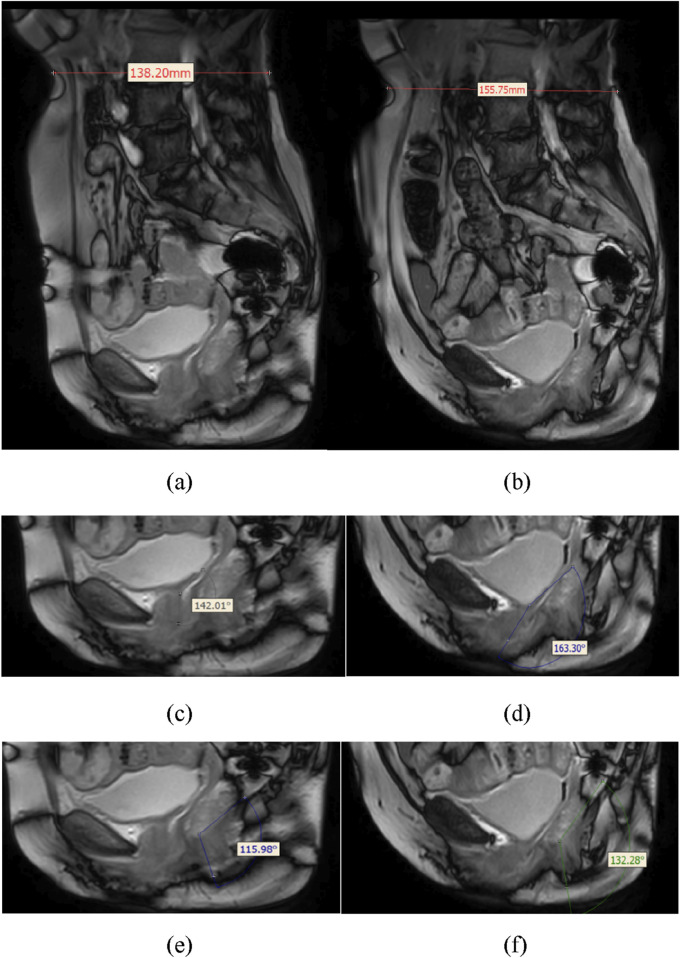
Schematic diagram showing anatomical measurements of the relative changes in waistline, RVA, and ARA from dynamic MRI in a human participant. **(a)** shows the waistline before Valsalva (138.20 mm), **(b)** shows the waistline after Valsalva (155.75 mm), **(c)** shows the RVA before Valsalva (142.01°), **(d)** shows the RVA after Valsalva (163.30°), **(e)** shows the ARA before Valsalva (115.98°), **(f)** shows the ARA after Valsalva (132.28°).

Figure 4 illustrates the validation of the model through dynamic MRI measurements during the Valsalva maneuver. These images demonstrate the anatomical changes induced by intra-abdominal pressure and provide quantitative references for validating the simulation model’s accuracy. Assessments of using data obtained from dynamic MRI of pelvic floor changes along the midsagittal plane in model during the Valsalva maneuver showed that the relative changes in abdominal waistline and the differences in RVA and ARA were within 8.28% (Table 4).

**TABLE 4:** Comparison of dynamic MRI and 3D model measurement results.

Validation indicators		Measurement results		Difference percentage
		Dynamic MRI	3D model	
Waistline	Before Valsalva	138.20 mm	135.81 mm	1.73%
	After Valsalva	155.75 mm	142.85 mm	8.28%
RVA	Before Valsalva	142.01°	140.80°	0.85%
	After Valsalva	163.30°	162.62°	0.42%
ARA	Before Valsalva	115.98°	116.32°	0.29%
	After Valsalva	132.28°	132.75°	0.36%

### 3.4 Finite element analysis results of simulation plans

**FIGURE 5:**
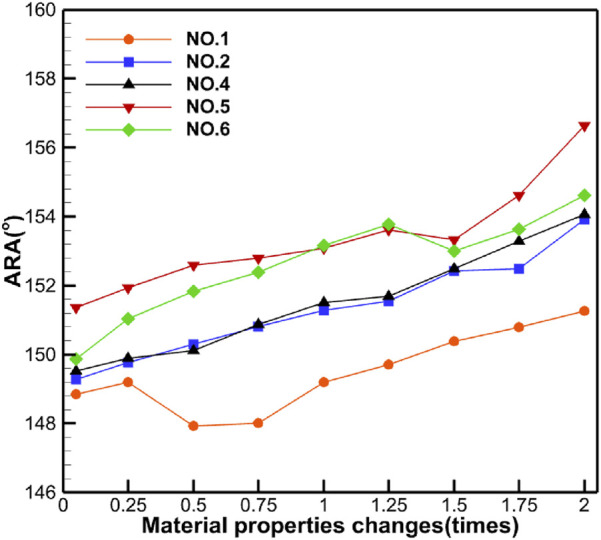
Curve chart of ARA changes with material properties. This figure shows the changes in ARA under different muscle intervention scenarios as the elastic modulus increases from 0.05 to 2 times the baseline value. Five muscle group combinations were simulated, including external anal sphincter; levator ani muscle; levator ani muscle and external anal sphincter; pelvic floor muscles; and pelvic floor muscles and rectus abdominis muscle and erector spinae muscle.

**FIGURE 6:**
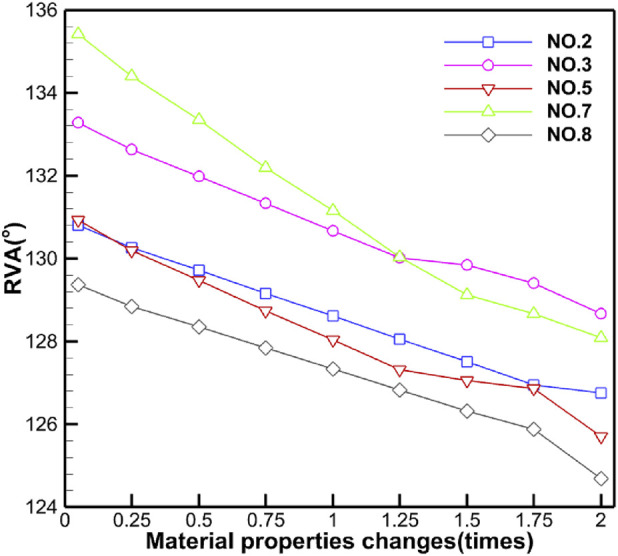
Curve chart of RVA changes with material properties. This figure shows the changes in RVA under different muscle intervention scenarios as the elastic modulus increases from 0.05 to 2 times the baseline value. Five muscle group combinations were simulated, including levator ani muscle; levator ani muscle and urethral sphincter; pelvic floor muscles; pelvic floor muscles and hip muscles; and pelvic floor muscles and rectus abdominis muscle and hip muscles and erector spinae muscle.

**FIGURE 7:**
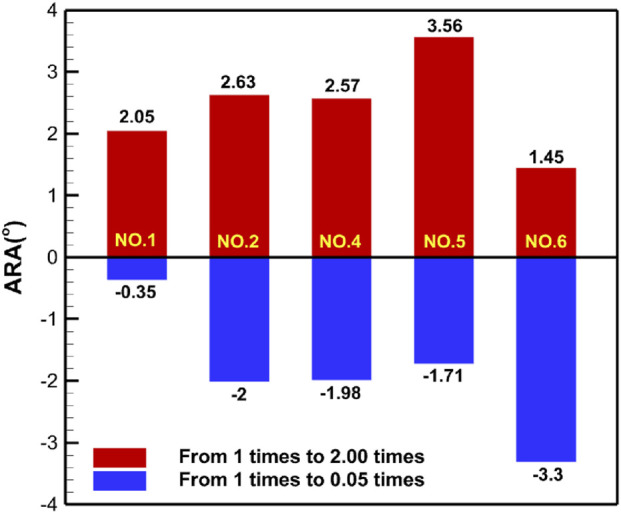
Absolute changes in ARA under different simulation plans for constipation rehabilitation and fecal incontinence rehabilitation. This figure presents the absolute changes in ARA under four constipation-related and fecal incontinence-related simulation plans. Condition No.1 simulates the external anal sphincter. Condition No.2 simulates the levator ani muscle. Condition No.4 simulates combined of the levator ani muscle and external anal sphincter. Condition No.5 simulates the pelvic floor muscles. Condition No.6 simulates combined of pelvic floor muscles and rectus abdominis muscle and erector spinae muscle. Blue bars indicate changes in ARA as muscle stiffness increases from 1 to 0.05 times the baseline, while red bars show changes from 1 times to 2 times.

**FIGURE 8:**
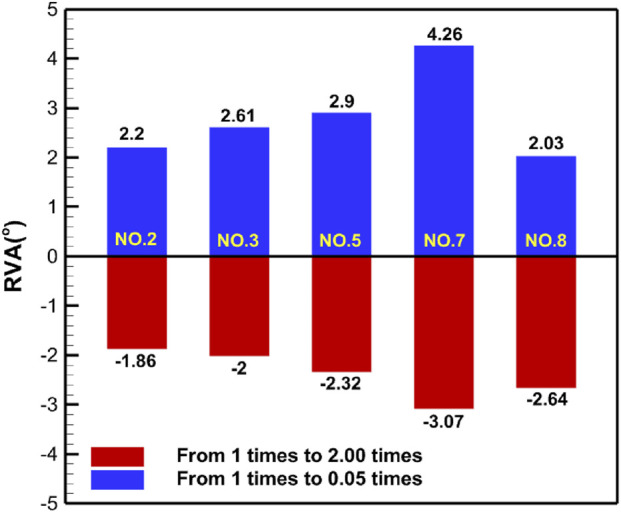
Absolute changes in RVA under different simulation plans for urinary incontinence rehabilitation. This figure presents the absolute changes in RVA under five urinary incontinence-related simulation plans. Condition No.2 simulates the levator ani muscle. Condition No.3 simulates combined of levator ani muscle and urethral sphincter. Condition No.5 simulates the pelvic floor muscles. Condition No.7 simulates combined of pelvic floor muscles and hip muscles. Condition No.8 simulates combined of pelvic floor muscles and rectus abdominis muscle and hip muscles and erector spinae muscle. Blue bars indicate changes in RVA as muscle stiffness increases from 1 to 0.05 times the baseline, while red bars show changes from 1 times to 2 times.

Table 5 shows the specific numerical changes in ARA and RVA as the material properties vary under different muscle combinations in the simulations of urinary and defecation dysfunction. Figures 5, 6 illustrate the trend of changes in ARA and RVA with varying material properties for different muscle combinations. Furthermore, as the material properties increase from 0.05 times to 2 times baseline, although the curves fluctuate, ARA shows an overall upward trend with a range of 147°∼157°, and RVA shows an overall downward trend with a range of 124°∼135°. Figures 7, 8 show the absolute changes in ARA and RVA under different conditions of simulated constipation, fecal incontinence, and urinary incontinence rehabilitation. Based on our previous research on elderly men (Wang et al., 2024b), we compared the order of muscles that benefit from rehabilitation training for urinary and defecation dysfunction in elderly men and elderly women (Table 6).

**TABLE 5:** Numerical table of ARA changes with material properties.

Conditions No.	Muscles	Angle	Types of urinary and defecation dysfunction	Multiple of material properties									Changes in material properties	
				0.05 times	0.25 times	0.50 times	0.75 times	1.00 times	1.25 times	1.50 times	1.75 times	2.00 times	From 1 times to 0.05 times	From 1 times to 2 times
No.1	External anal sphincter	ARA	Fecal incontinence	148.85	149.2	147.93	148	149.2	149.71	150.38	150.79	151.25	0.35	2.05
No.2	Levator ani muscle	ARA	Constipation, fecal incontinence	149.28	149.77	150.29	150.8	151.28	151.54	152.42	152.49	153.91	2	2.63
No.4	Levator ani muscle and external anal sphincter	ARA	Constipation	149.52	149.88	150.11	150.87	151.5	151.68	152.49	153.29	154.07	1.98	2.57
No.5	Pelvic floor muscles	ARA	Constipation, fecal incontinence	151.37	151.93	152.58	152.79	153.08	153.62	153.32	154.62	156.64	1.71	3.56
No.6	Pelvic floor muscles and rectus abdominis muscle and erector spinae muscle	ARA	Fecal incontinence	149.87	151.04	151.84	152.39	153.17	153.77	153	153.63	154.62	3.3	1.45
No.2	Levator ani muscle	RVA	Urinary incontinence	130.81	130.26	129.71	129.16	128.61	128.05	127.51	126.95	126.75	2.2	1.86
No.3	Levator ani muscle and urethral sphincter	RVA	Urinary incontinence	133.28	132.63	131.98	131.33	130.67	130.02	129.84	129.41	128.67	2.61	2
No.5	Pelvic floor muscles	RVA	Urinary incontinence	130.93	130.2	129.47	128.74	128.03	127.32	127.05	126.86	125.71	2.9	2.32
No.7	Pelvic floor muscles and hip muscles	RVA	Urinary incontinence	135.42	134.4	133.34	132.19	131.16	130.04	129.13	128.66	128.09	4.26	3.07
No.8	Pelvic floor muscles and rectus abdominis muscle and hip muscles and erector spinae muscle	RVA	Urinary incontinence	129.36	128.85	128.35	127.84	127.33	126.82	126.31	125.88	124.69	2.03	2.64

**TABLE 6:** Comparison of the muscle prioritization of rehabilitation training for urinary and defecation dysfunction between elderly men and women.

Urinary and defecation dysfunction	Gender	Material properties	The muscle prioritization for rehabilitation training
Constipation	Elderly men	From 1 times to 0.5 times	Levator ani muscle
		From 1 times to 2 times	Levator ani muscle + external anal sphincter
	Elderly women	From 1 times to 0.5 times	Levator ani muscle
		From 1 times to 2 times	Pelvic floor muscles
Fecal incontinence	Elderly men	From 1 times to 0.5 times	Levator ani muscle
		From 1 times to 2 times	External anal sphincter
	Elderly women	From 1 times to 0.5 times	Pelvic floor muscles + rectus abdominis muscle+erector spinae muscle
		From 1 times to 2 times	Pelvic floor muscles
Urinary incontinence	Elderly men	From 1 times to 0.5 times	Levator ani muscle
		From 1 times to 2 times	Pelvic floor muscles
	Elderly women	From 1 times to 0.5 times	Pelvic floor muscles+hip muscles
		From 1 times to 2 times	Pelvic floor muscles+hip muscles

## 4 Discussion

The study targets a highly relevant issue in elderly women—urinary and fecal dysfunction—and proposes personalized rehabilitation guidance using biomechanical modeling. Compared with previous studies that focused solely on pelvic floor muscles, this research firstly developed a comprehensive 3D pelvic-thigh model in elderly women. The findings highlight the critical role of integrating muscle groups beyond the pelvic floor, including the abdominal, hip, and back muscles, in maintaining control of urination and defecation and achieving functional recovery. This comprehensive approach enhances the practical relevance of the study.

In constructing the present model, a healthy elderly female dataset was deliberately selected to provide a geometrically intact baseline for biomechanical simulation. In this study, the elastic modulus of muscles was proportionally adjusted to approximate rehabilitation-induced adaptations within a finite element framework. Studies have shown that the elastic modulus is associated with neuromuscular control (Dickson et al., 2024; Wolff et al., 2020), and rehabilitation training not only alters the active contractile capacity of muscles but also induces passive mechanical changes, which can be observed at both the single-fiber and fiber-bundle levels (Noonan et al., 2020; Kawama et al., 2024). In addition, oportional modulation of material parameters has also been adopted in previous FEA studies (Dias et al., 2017), supporting its feasibility as an exploratory strategy.

The model extends beyond traditional pelvic floor simulations because it was constructed based on data from dynamic MRI, CT, and static MRI data, and its accuracy was validated through geometric comparison with key anatomical landmarks, including the waistline, RVA, and ARA. While the model showed good geometric agreement with key pelvic floor anatomical structures, the observed waistline variation of 8.28% was slightly higher than anticipated. This discrepancy can be attributed to the complex nature of soft tissue deformation, which may explain the larger variation observed in waist circumference compared to ARA and RVA. The resulting model showed good geometric agreement with key pelvic floor anatomical structures in a representative elderly female, supporting its preliminary applicability in simulating pelvic biomechanics. These findings suggest potential utility for understanding mechanisms underlying urinary and fecal control, although broader validation is needed.

The study employed RVA and ARA as the sole indicators for assessing urinary and fecal control. At rest, the puborectalis muscle continuously pulls the rectum forward to maintain an appropriate ARA (Zhou and Lai, 2021), which generally ranges from 90° to 127°, and normally increases to 120°∼152.4°during defecation (Zhou and Lai, 2021; Kobi et al., 2018; MR Group, 2022). Failure of the ARA to increase, or paradoxical narrowing during defecation, indicates pelvic floor muscle relaxation, loss of tone, or impaired coordination. Conversely, when weakening of the puborectalis muscle leads to an ARA exceeding the normal range, fecal incontinence may occur, often associated with reduced pelvic floor activity or pudendal nerve injury (Elmalı, et al., 2024). RVA reflects the integrity of bladder neck closure, with a normal resting range of 90°∼120° (Liu et al., 2013). In pelvic floor dysfunction, the RVA may abnormally widen to 160°∼180°during straining (Fielding et al., 2000), and in patients with stress urinary incontinence, the RVA often exceeds 140° (Li and Yan, 2015), suggesting decreased urethral closure pressure and impaired urinary continence. The results showed that as the muscle material coefficients gradually increased from 0.05 times to 1 times or decreased from 2times to 1 times, both RVA and ARA values progressively approached the normal range. These biomechanical changes suggest a potential mechanism whereby targeted reinforcement of the pelvic floor, abdominal, hip, and back muscles through rehabilitation interventions may enhance pelvic floor functional capacity and thereby improve urinary and fecal dysfunction.

While RVA and ARA are significant imaging biomarkers, urinary and defecation dysfunction are complex clinical symptoms whose severity is closely linked to patient subjective experience, quality of life, and neural control. Alterations in these two parameters alone do not fully capture the comprehensive clinical functional enhancement. In this study, RVA and ARA served as the primary outcome measures because they are the most readily quantifiable biomechanical indicators within the finite element modeling framework. Thus RVA and ARA should be interpreted as surrogate biomechanical markers rather than comprehensive clinical endpoints.

For the first time, this study provides preliminary evidence on the potential muscle prioritization of pelvic floor rehabilitation benefits for elderly women. For constipation management, computational findings suggest that rehabilitation targeting the levator ani muscle might be prioritized when muscle properties fall below baseline levels (1 time), while cases with above-baseline muscle properties may benefit more from comprehensive pelvic floor muscle engagement. Regarding fecal incontinence, sub-baseline muscle conditions suggest the need for integrated training targeting pelvic floor muscles along with rectus abdominis, and erector spinae muscles, whereas supra-baseline conditions may warrant concentrated pelvic floor muscle rehabilitation. For urinary incontinence cases, combined pelvic-hip muscles rehabilitation appears advantageous regardless of baseline muscle status. These observed associations between muscle material coefficients and rehabilitation outcomes highlight the importance of condition-specific muscle prioritization in elderly women’s pelvic rehabilitation, these findings may contribute to the development of more tailored rehabilitation nursing recommendations. It should be emphasized that our interpretation focused on the general trends observed when material properties were below or above baseline, rather than the extreme values of parameter variation. The broader range (0.05–2.0) was applied primarily to test model robustness, while clinical rehabilitation is unlikely to reach such extremes.

Recent clinical research by Ojukwu et al. (2022) demonstrated that hip exercises alone, as well as combined abdominal, hip, and pelvic floor muscles training, significantly enhance pelvic floor muscle activity. This may be attributed to the synergistic contractions induced by adductor and gluteal muscle engagement, which facilitate the co-contraction of pelvic floor muscles and the striated urethral sphincter, thereby strengthening pelvic floor muscles contraction and improving neuromuscular control (Marques et al., 2020). These findings are consistent with our simulation-based results, which suggest that pelvic floor-hip combined training may offer more comprehensive benefits for elderly women with urinary incontinence. In contrast, clinical and imaging studies directly addressing the prioritization of specific muscle groups in constipation and fecal incontinence rehabilitation are limited. Currently, research on pelvic floor muscle training (PFMT) for elderly women primarily focuses on the overall efficacy of PFMT, rather than the sequence or prioritization of individual muscle group interventions. As such, direct clinical comparisons with the prioritization patterns observed in our simulations remain exploratory.

Compared to our previous findings (Wang et al., 2024b), there were notable differences in the key muscles for rehabilitation of urinary and defecation dysfunction between elderly men and women. In elderly men, the levator ani muscle and external anal sphincter are the critical muscles for improving defecation dysfunction. In contrast, for elderly women, the levator ani, pelvic floor, rectus abdominis, and erector spinae muscles play more significant roles in improving defecation dysfunction. In terms of urinary incontinence, for elderly men, training should primarily focus on the levator ani and pelvic floor muscles, while for women, combined training of the pelvic floor muscles and hip muscles, or a comprehensive approach targeting the pelvic floor muscles, rectus abdominis muscle, hip muscles, and erector spinae muscle, is more effective in restoring urinary control. Specifically, rehabilitation training for urinary and defecation dysfunction in elderly men focuses on strengthening localized muscles, such as the levator ani muscle and external anal sphincter, and the benefits of such interventions are concentrated on localized improvements in the core muscle groups. In contrast, rehabilitation training for elderly women emphasizes the synergistic action of multiple muscle groups, focusing on overall functional recovery. Compared to their male counterparts, the neuromuscular system in elderly women undergoes more severe degeneration (Stalling et al., 2024). The decline in estrogen levels leads to a reduction in neuromuscular function in postmenopausal women, negatively affecting muscle coordination (Willoughby et al., 2024; O'Bryan et al., 2025). Studies have shown that while elderly men tend to exhibit greater muscle size, strength, and functional performance, elderly women demonstrate a smaller cross-sectional area of the lateral thigh muscle, lower knee extensor torque, and poorer force steadiness in muscles around the hip joint (Guo et al., 2023). Anatomically, the pelvic floor muscles in women are more vulnerable, as they are heavily influenced by pregnancy and childbirth. Therefore, rehabilitation training for elderly women requires special attention to synergistic training of the muscles of the pelvic floor, abdomen, hip, and back, ensuring a comprehensive approach to improve their functionality.

These findings suggest that sex-based differences may influence muscle prioritization in pelvic floor rehabilitation, underscoring the value of further investigation into gender-specific rehabilitation strategies. Clinical phenomena also confirm that gender differences lead to different types of diseases in patients. In clinical practice, elderly men are more prone to defecatory dyssynergia, whereas women are more likely to experience insufficient defecatory propulsion (Abe et al., 2023). Women are also more susceptible to stress urinary incontinence, while men more commonly present with urge urinary incontinence (Hu et al., 2024). Bjarnason-Wehrens et al. (2007) proposed in 2007 that sex-based issues should be considered in cardiac rehabilitation, recommending customizing programs specifically for women with ischemic heart disease. Similarly, the anatomical and functional differences between men and women must be fully considered to provide more targeted rehabilitation plans for elderly patients urinary and defecation dysfunction, ultimately enhancing quality of life and functional recovery.

While this computational evidence provides mechanistic insights, validating the proposed muscle prioritization strategies in real-world settings through controlled clinical trials is essential. Shear wave elastography (SWE), an ultrasound-based elastography technique that measures tissue elasticity modulus by generating shear waves within the tissue via external or internal forces and analyzing their propagation speed (Li, 2017). With a penetration depth exceeding 8 cm (Frulio and Trillaud, 2013), SWE provides critical technical support for identifying muscle fibrosis, damage, or excessive laxity, especially in deep pelvic structures. It is non-invasive, repeatable, and has shown potential in evaluating muscle stiffness and contractility in the levator ani (Xie et al., 2015), puborectalis (Wang et al., 2017), and other pelvic floor muscles (Okada et al., 2001; Tang et al., 2020). Studies suggest that SWE can guide early pelvic floor muscle training and monitor rehabilitation outcomes (Zhou M. et al., 2020; Jiang et al., 2019; Bai et al., 2022; Niu et al., 2018). Future research should employ SWE to quantitatively assess the elastic modulus of pelvic floor-related muscles in patients with urinary and fecal dysfunction. Building on the muscle prioritization trends observed in our model, future research may explore constructing personalized rehabilitation protocols that consider individual muscle characteristics, sex differences, and dysfunction types. Subsequently, the effectiveness of these protocols will be evaluated using functional outcomes such as reduction in urinary and fecal dysfunction symptoms, ARA, RVA, and quality of life. This two-stage validation—biomechanical characterization followed by targeted intervention—will help bridge the gap between simulation results and clinical application.

Striking an appropriate balance between model fidelity and practical feasibility remains a critical and ongoing challenge (Moseley et al., 2025). It is important to acknowledge certain limitations of this study. First, in this study, muscle elastic modulus was linearly scaled to represent rehabilitation-induced functional improvement. This approach, while a common simplification in finite element modeling, primarily reflects changes in passive stiffness rather than improvements in active contraction capacity or neuromuscular coordination. As such, our method more closely simulates muscle dynamic alterations such as fibrosis than genuine training-induced strengthening.

In reality, rehabilitation induces complex bioadaptive processes that include neuromuscular control, motor unit recruitment, muscle fiber type conversion, and myofascial coordination, which cannot be fully represented by a single parameter such as passive stiffness. Therefore, our finite element results should be interpreted as reflecting the structural potential of muscle strengthening, rather than capturing the entire spectrum of functional adaptations. Future studies combining finite element modeling with electrophysiological measurements and clinical outcomes are needed to provide a more comprehensive understanding.

Second, the boundary conditions were idealized, with bones being modeled as completely fixed, rigid bodies and intra-abdominal pressure assumed to be uniformly distributed. Compared with soft tissues, the elastic modulus of bone is much higher, and thus its deformation can be considered negligible relative to that of soft tissues. Therefore, bone was simplified as a rigid body in this study. Furthermore, our dynamic MRI measurements indicated that bone displacement during rehabilitation training was minimal, and accordingly, the bones were simplified as fully fixed. *In vivo*, however, pelvic floor mechanics involve micro-articulations, pressure vessel effects, and holistic load transmission. In future work, we plan to incorporate such dynamic interactions to improve the accuracy of the computational model.

Third, although the constructed model provides valuable biomechanical insights, the modeling was based on a single elderly female participant, which inevitably limits the generalizability of the findings. BMI, individual anatomical differences, and tissue properties may alter biomechanical outcomes. To improve generalizability and clinical applicability, future studies should involve multidisciplinary collaboration to develop multi-subject models incorporating diverse anatomical and clinical characteristics.

Fourth, material properties were varied linearly from 0.05 to 2.0 times baseline to represent functional impairment and enhancement. The analysis primarily contrasted sub-baseline (<1.0) and supra-baseline (>1.0) conditions to describe directional trends, rather than drawing predictions from the extreme ends of the range. Nevertheless, the assumption of linear extrapolation across such a wide interval does not fully reflect biological reality, since muscle performance usually operates within an optimal functional range. Therefore, results at extreme values should be regarded as theoretical boundaries.

The findings of this study are primarily theoretical recommendations derived from computational simulations without clinical validation, direct clinical implementation of its findings risks premature translation. Future research should involve designing studies to evaluate the feasibility and efficacy of these muscle prioritization in real-world elderly population. We are currently conducting a larger-scale SWE-based clinical validation to bridge simulation with real-world applications. Despite these limitations, the present model provides novel mechanistic insights into the interplay of pelvic and extra-pelvic muscles in continence control. This study represents the first attempt to integrate pelvic floor, abdominal, hip, and back muscles into a unified model for elderly women, providing valuable biomechanical insights and a foundation for precision rehabilitation and AI-driven prescription systemsin pelvic floor care.

## 5 Conclusion

The study developed a 3D finite element model that is highly consistent with the actual pelvic floor conditions of an elderly woman and validated its effectiveness using dynamic MRI data. By employing FEA, this study delved into the mechanisms underlying the enhancement of urinary and defecation control in the elderly women and identified the differing muscle prioritization for rehabilitation training in elderly men and women. These findings facilitate the personalized recommendation of pelvic floor rehabilitation plans tailored to the muscle condition and dysfunction type of elderly women. The current simplified model was developed from a single elderly female subject, which limits its generalizability. Therefore, it is imperative that future work should focus on developing multi-subject and multi-sample models that account for anatomical and physiological variability. Additionally, clinical validation trials should be undertaken to ensure the broader applicability of rehabilitation strategies.

## Data Availability

The raw data supporting the conclusions of this article will be made available by the authors, without undue reservation.
