# Supplementary material for: Finite element-based prioritization of pelvic floor muscles for rehabilitation to maintain urinary and fecal control in elderly women
Source: Front Physiol. 2025 Nov 24;16:1663545. doi: 10.3389/fphys.2025.1663545 (PMC12682634; doi:10.3389/fphys.2025.1663545)
Supplement: Supplementary file 1 [file Supplementaryfile1.docx]

**
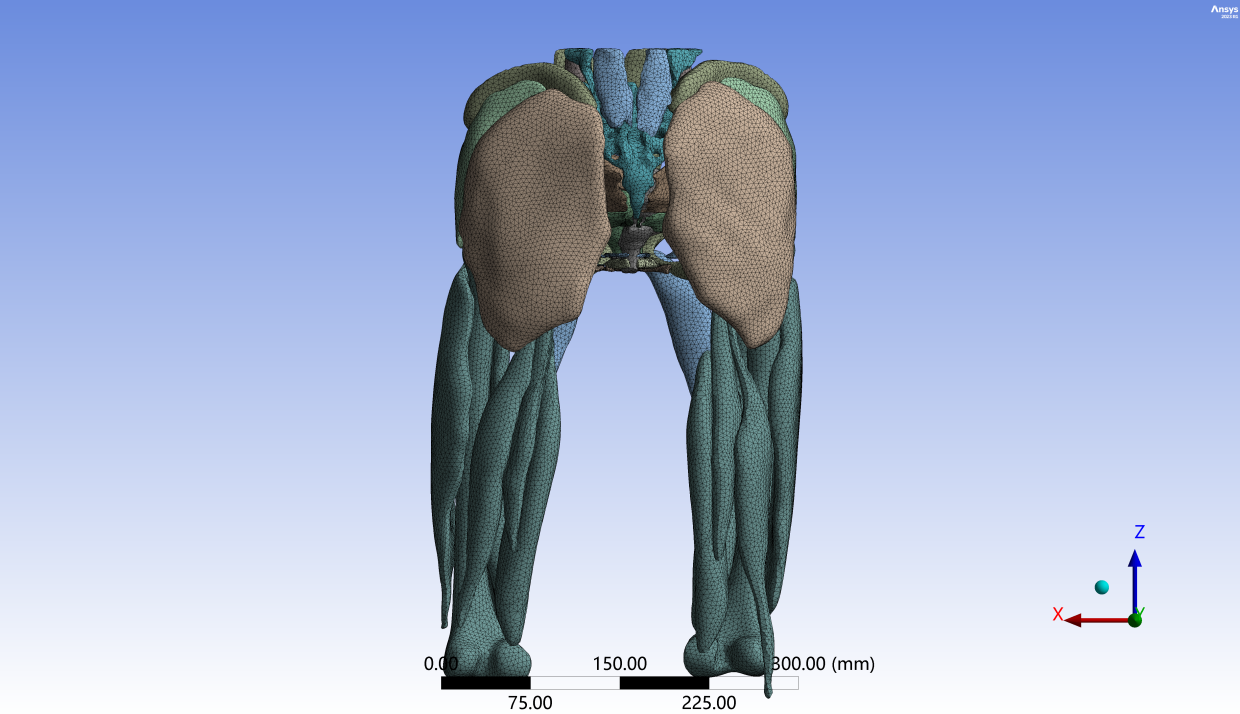
**

**Figure S1 Mesh Distribution of Pelvic Floor Model with Initial Mesh Density**

**
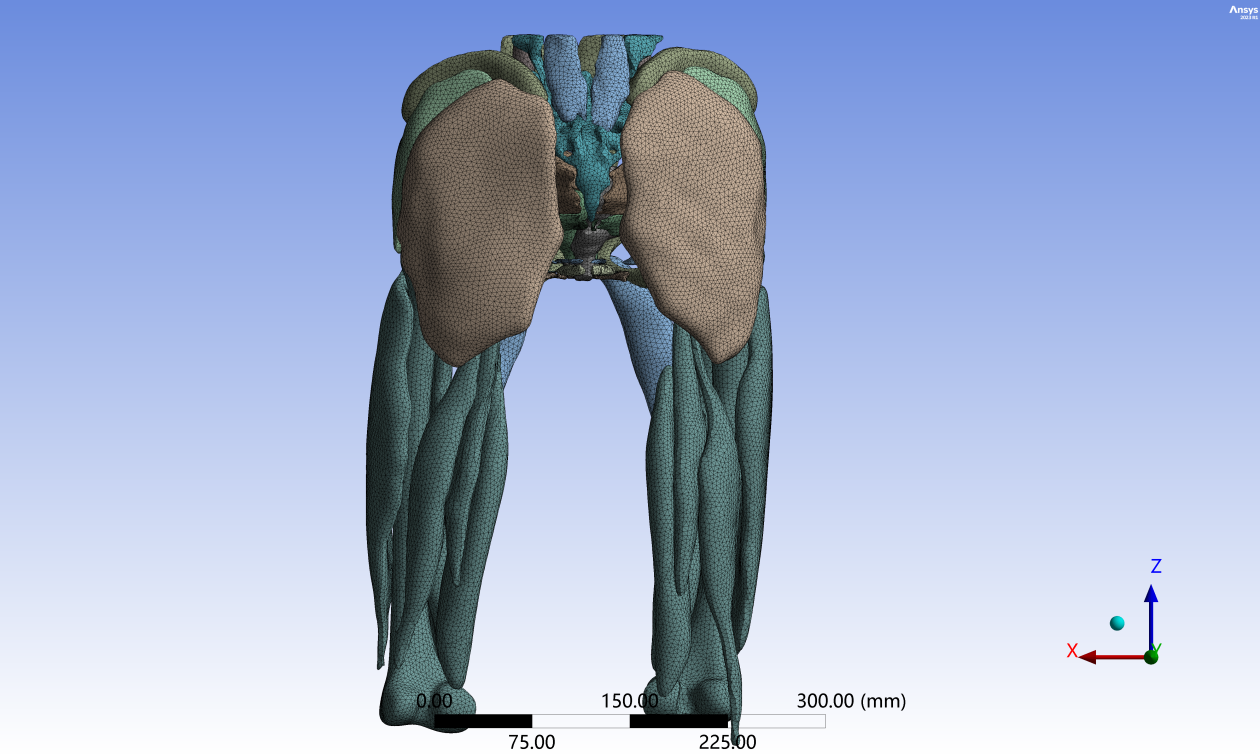
**

**Figure S2 Mesh Distribution of Pelvic Floor Model with Refined Mesh Density**

**
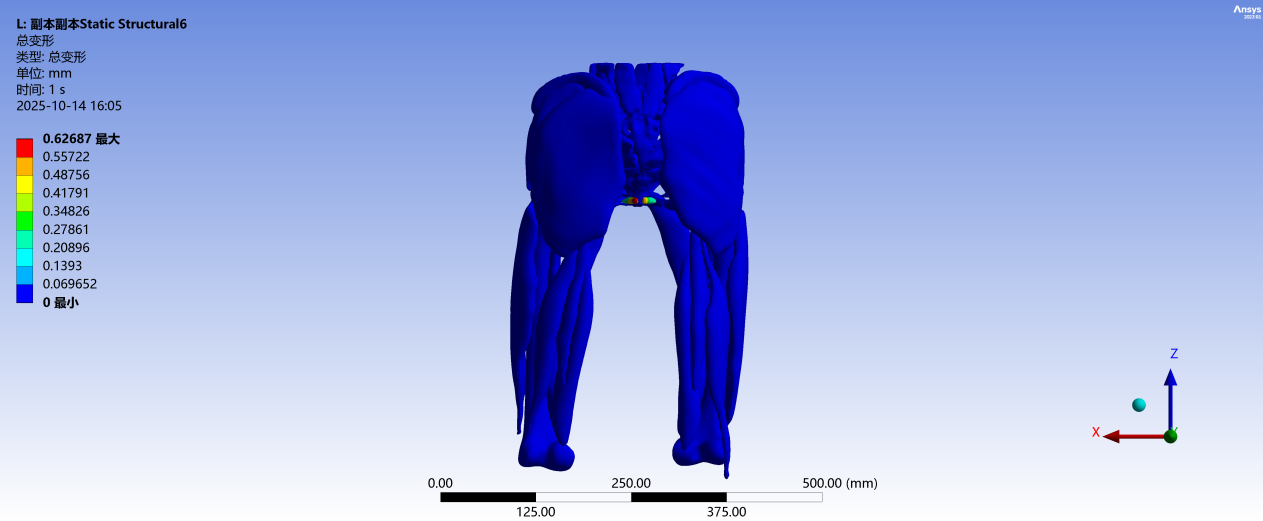
**

**Figure S3 Total Deformation Distribution for 100% Mesh Density Model**

**
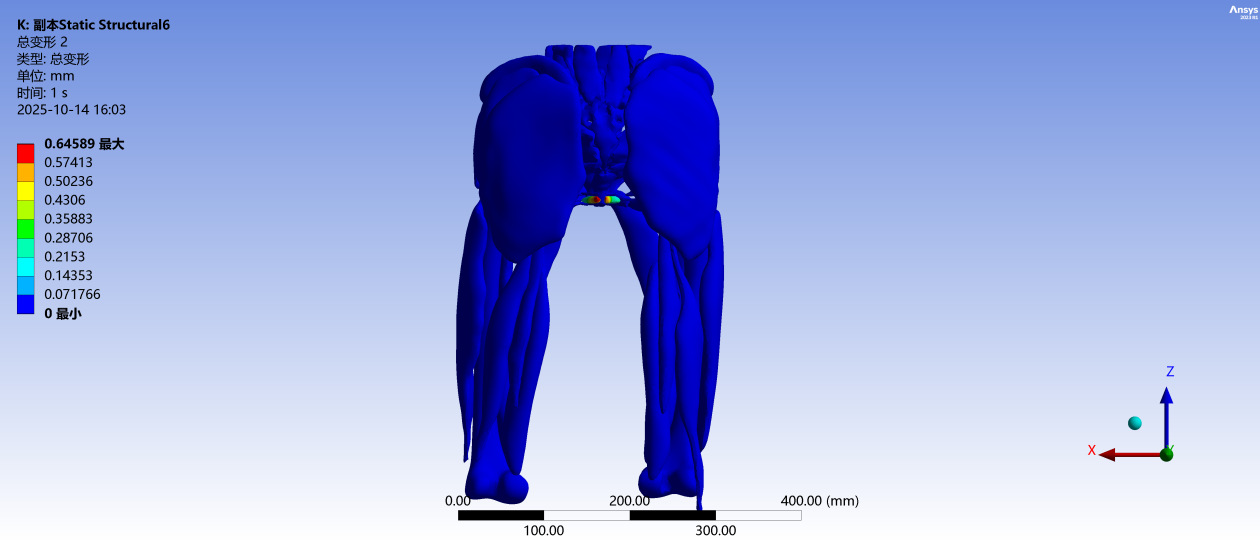
**

**Figure S4 Total Deformation Distribution for 130% Mesh Density Model**
